# Early and Midterm Outcomes of the VSSR procedure with De Paulis valsalva graft: A Chinese single-Center Experience in 38 patients

**DOI:** 10.1186/s13019-015-0347-1

**Published:** 2015-11-19

**Authors:** Lili Xu, Feng Gao, Ping Li, Yi Xu, Shuo Liu, Bing You, Li-Zhong Sun

**Affiliations:** Department of Cardiovascular Surgery, Beijing Institute of Heart, Lung and Blood Vessel Diseases, Beijing Anzhen Hospital, Capital Medical University, 2 Anzhen Road, Beijing, 100029 China

**Keywords:** Valve-sparing aortic root replacement, De Paulis graft VSSR procedure

## Abstract

**Background:**

This study investigated early and midterm outcomes after valve-sparing aortic root replacement (VSSR procedure with De Paulis Valsalva graft) for acute aortic dissection or ascending aortic aneurysm in a single Chinese hospital center.

**Methods:**

Between September 2005 to July 2013, 38 patients (84.2 % male; mean age, 45.5 ± 12.4) underwent VSSR procedure with De Paulis valsalva graft for acute aortic dissection or ascending aortic aneurysm and were followed up clinically and echocardiographically.

**Results:**

Among the 38 cases studied, intensive care unit stay duration was 34.5 (interquartile range, 16–34.6) days; hospital stay duration was 11.7 ± 7.9 days; operation time was 6.8 ± 1.9 h; and cross-clamping time was 154.4 ± 42.0 min. There was one intraoperative conversion to Bentall procedure; one re-operation for bleeding; one operative death and one case who developed complications. Mean follow-up was 39.7 ± 21.7 months (range, 12–108 months; cumulative rate, 1483 patients-months; follow-up rate, 94 %). At 5 and 10 years, overall freedom from valve replacement was 94 % and 87 %; freedom from aortic regurgitation grade II or higher was 94 % and 91 %; and freedom from reoperation was 94 % and 90 % years, respectively.

**Conclusions:**

The reimplantation type of valve-sparing procedure appears to be facilitated by the use of the De Paulis valsalva graft with satisfactory perioperative and midterm results.

## Background

Until the introduction of valve-sparing aortic root replacement (VSSR) in 1992 [1], acute aortic dissection or ascending aortic aneurysm was traditionally treated by replacing the root with a composite valve graft [2], which was limited by patient’s postoperative quality of life and lifelong dependency on anticoagulation. VSSR has been reported to be associated with favorable long-term outcomes and mortality [3]. We report here on the short- and midterm outcomes of 38 patients who underwent VSSR with De Paulis Valsalva graft for acute aortic dissection or ascending aortic aneurysm and were followed up for up to 9 years at our center in China.

## Methods

From September 2005 to July 2013, 38 patients (32 males, 84.2 %; mean age, 45.5 ± 12.4; range, 24–69 years) underwent VSSR procedure at Anzhen Hospital, Beijing. As summarized in Table 1, mean age of the overall cohort (*n* = 38) was 45.5 ± 12.4 years (range, 24–69); 84.2 % were male; mean body mass index (BMI) was 21.78 ± 8.4; 18 (47.4 %) patients were greater than NYHA class 3; EuroScore II was 13.9 (IQR, 1.83–23.3); 22 (57.9 %) patients had greater than moderate aortic valve regurgitation; 7 (18.4 %) patients had Marfan syndrome and LDS; and 16 (42.1 %) had aortic dissection. Following guidelines [4], asymptomatic patients with an aortic root diameter of more than 55 mm due to an aneurysm of degenerative origin were selected for operation. If the patient had been diagnosed with Marfan or LDS, VSSR procedure was indicated when the aortic root diameter exceeded 45 mm. VSSR was performed in patients with acute aortic dissection if the aortic root was destroyed regardless of the root diameter. This study was approved by the Regional Ethics Committee of our hospital and all patients signed informed consents.

**Table 1:** Preoperative characteristics (*n* = 38)

Variables	Values
Age, years (range)	45.5 ± 12.4 (24–69)
Male gender	32 (84.2 %)
Female gender	6 (15.8 %)
Weight, Kg	73.9 ± 12.3
Height, cm	173.7 ± 9.3
BMI	21.78 ± 8.4
BSA, m^2^	1.97 ± 0.2
NYHA classification	
Class I	0
Class II	20
Class III	15
Class IV	3
Hypertension	15
Chronic obstructive pulmonary disease	3
Smoking	13
Peripheral vascular disease	3
Preoperative carnine	86.5 ± 24.0
Preoperative atrial fibrillation or flutter	1
EuroScore II	13.9 (IQR, 1.83–23.3)
Preoperative UCG	
LVEF	64.0 ± 6.5
Maximum aortic arch diameter, mm	31 ± 5.9
Maximum aortic root diameter, mm	47.5 ± 11.0
Maximum diameter, mm	48.9 ± 11.1
Sinotubular junction diameter, mm	45.0 ± 9.9
Maximum aortic annulus diameter, mm	25.8 ± 2.7
Aortic valve regurgitation	
None	8
Mild	4
Moderate	6
Moderately severe	3
Severe	13
Mitral regurgitation	13
Tricuspid regurgitation	7
Marfan syndrome and LDS	7
Non-connective tissue disease	31
Aneurysm without dissection	22
Acute dissection	16
DeBakey I	14
DeBakey II	2
Bicuspid aortic valve	2

### Surgical technique [5]

All procedures were performed through a median sternotomy using cardiopulmonary bypass. Patients undergoing isolated aortic root replacement received right atrial two-stage and central aortic cannulation. In most cases, antegrade cerebral perfusion was used throughout the period of hypothermic systemic circulatory arrest. After ascending aorta was clamped, the aorta was then transected, the aortic cusps were carefully inspected for prolapse, tears, calcification and perforations. After careful assessment of pertinent parameters to determine VSSR procedure methodology, the root was circumferentially dissected down to the aortic annulus nadir and each coronary artery was sutured as a button, freed and mobilized. A De Paulis valsalva graft (Gelweave Valsalva graft, Vascutek Ltd, Renfrewshire, Scotland) was secured to the aortic annulus and passed on a horizontal plane below the aortic cusps. Three aortic junction crests were anchored with 4–0 prolene suture inside the valsalva graft and throughout the graft wall up to the height mark. The aortic annulus then were sutured inside the valsalva graft using interrupted 2–0 polypropylene horizontal mattress sutures, passed though the wall of the graft and tied on the outside. The graft was re-implanted on the left ventricular outflow, and the coronary buttons then were re-implanted on the graft using 6–0 polypropylene. The distal anastomosis of the graft was continuously sutured to the aorta with 4–0 prolene (in aortic arch replacement, it was sutured to the other graft) (Table 2).

**Table 2:** Perioperative data (*n* = 38)

Variable	
Operation time, hours	6.8 ± 1.9
Cross-clamping time, minutes	154.4 ± 42.0
Bypass time, minutes	203.1 ± 60.1
Ventilation time, hours	35.2 ± 29.8
Intensive care unit stay duration, hours	34.5 (IQR, 16–34.6)
Postoperative hospital stay duration, days	11.7 ± 7.9
Additional procedures	
Coronary artery bypass grafting	4
Mitral valve replacement	1
ASD or VSD closure	4
Arch and “Trunk” procedure	16
Postoperative Complications	
Postoperative arrhythmias	11
Thoracotomy hemostasis	1
Surgical infections	3
Psychiatric symptoms	5
Acute renal failure	3
Postoperative drainage, mL	1201.3 ± 757.7
Death	1

### Follow-up

**Fig. 1:**
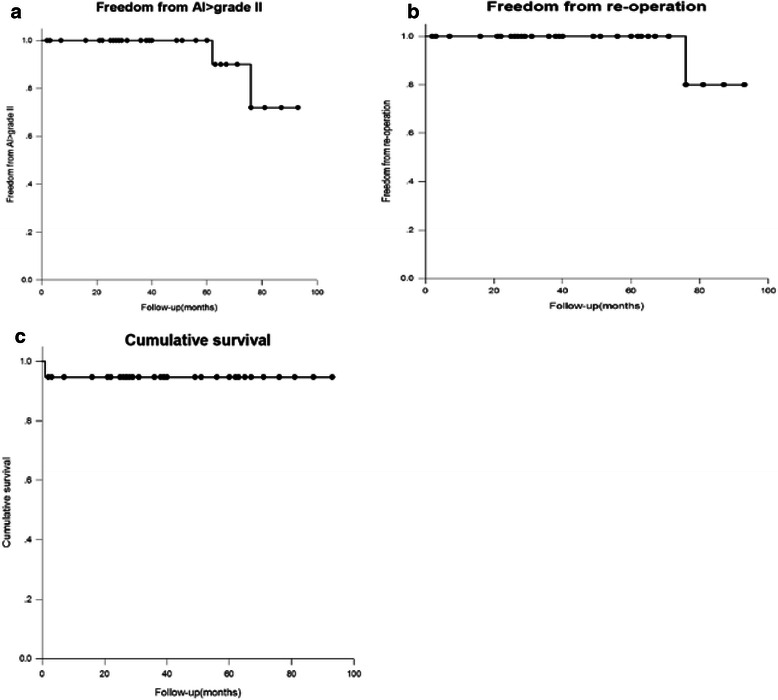
Kaplan-Meier analysis of **a** freedom from aortic insufficiency > grade 2; **b** freedom from reoperation; and **c** cumulative survival

**Fig. 2:**
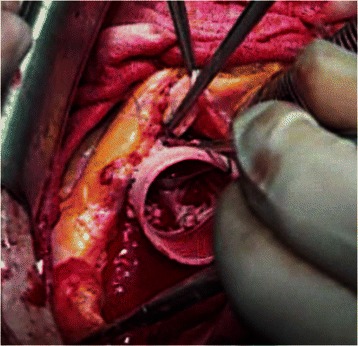
VSSR procedure with De Paulis Valsalva graft

Early death was defined as that occurring within 30 days of the operation. Calculation of postoperative hospital stay duration did not include early death cases. The median duration of follow-up for survivors was 39.7 ± 12.7 months (range, 12–108 months). To obtain follow-up information, clinical staff would first contact patients via phone. If patient could not be reached by telephone, letters were sent to the patient’s address. No patient was lost to follow-up in the early stage. Follow-up echocardiography was performed and evaluated for all patients. Postoperative death and repair failure requiring reoperation (severe aortic regurgitation, endocarditis and graft infection) after VSSR were defined as adverse events. Intraoperative conversion to a valve-replacing procedure due to severe valve regurgitation also was considered an adverse event (Figs. 1 and 2).

### Statistical methods

Data are presented as number and percentage for all discrete variables. Continuous variables are presented as mean ± standard deviation or median and interquartile range. Group comparisons were performed using the student t test for normally distributed variables, the Mann–Whitney U test for ordinal variables, and the Fisher exact test for categorical variables. A *p* value less than 0.05 was considered statistically significant. Overall survival and freedom from adverse events statistics were estimated with the Kaplan-Meier survival model. Data were analyzed with a commercially available software (SPSS Statistics 21; SPSS, IBM, New York, NY).

## Results

### Perioperative outcome

The VSSR procedure was performed in 37 patients, and one patient underwent intraoperative conversion to Bentall procedure. Within 30 days, there was one (2.6%) death secondary to lung infection coupled with multiple organ failure; 16 (42.1 %) emergency operations; and one (2.6 %) patient required re-exploration for bleeding. Additional procedures included CABG in 4 (10.5 %) patients; and arch and “trunk” procedure in 16 (42.1 %) patients. Five (13.2 %) patients developed psychiatric symptoms, and 3 (7.9 %) acute renal failure.

Mean duration of intensive care unit stay was 34.5 (IQR, 16–34.6) hours; and mean hospital stay was 11.7 ± 7.9 days. At discharge, mean operation time was 6.8 ± 1.9 h, and mean cross-clamping time was 154.4 ± 42.0 min. Aortic regurgitation less than grade I was documented echocardiographically in all 37 patients (one conversion to Bentall procedure) after the VSSR procedure, a significant reduction from the baseline of 20 (*p* < 0.001, Table 3).

**Table 3:** Follow-up data

Variables	Pre-op (*n* = 38)	Hospital discharge (*n* = 37)	1 year follow-up (*n* = 27)	5 years follow-up (*n* = 12)	*P* value 1	*P* value 2	*P* value 3
Aortic annulus diameter	25.8 ± 2.7	27.6 ± 1.9	27.8 ± 2.4	28.0 ± 1.9	<0.001	0.884	0.769
Aortic sinus diameter	48.9 ± 11.1	31.8 ± 3.1	30.8 ± 2.7	33.5 ± 3.3	<0.001	0.544	0.875
Aortic regurgitation (moderate to severe)	20	0	1	2	<0.001	0.226	<0.001
LVEF	64.0 ± 6.5	63.4 ± 6.5	63.2 ± 6.1	61.5 ± 6.6	0.634	0.769	0.560
LV end-systolic diameter	34.4 ± 5.6	31.9 ± 4.8	32.4 ± 2.9	31.5 ± 3.6	<0.001	0.812	0.661
LV end-diastolic diameter	53.7 ± 6.1	48.4 ± 5.5	48.7 ± 4.0	49.4 ± 4.9	<0.001	0.793	0.724

### Midterm follow-up

During mean follow-up time of 39.7 + 12.7 months (range, 12–108 months; cumulative rate, 1483 patient-months; and follow-up rate, 94 %), there was one death (2.7 %) secondary to lung infection within one year; one patient (2.7 %) experienced worsening of aortic regurgitation requiring reoperation and underwent aortic valve replacement; and the degree of regurgitation remained constant in most patients.

At 1 and 5 years, overall freedom from valve replacement was 94 % and 87 %; freedom from aortic regurgitation grade II or higher was 94 % and 91 % (included the one patient re-operated for aortic insufficiency); and freedom from reoperation was 94 % and 90 % years, respectively. Freedom from reoperation in patients without additional cusp repair was 92 % and 87 % at 1 and 5 years, respectively, which was not significantly different from those patients who required additional cusp repair (freedom from reoperation of 100 % at 5 years, *P* = 0.211).

## Discussion

As an alternative method to Bentall operation for acute aortic dissection or ascending aortic aneurysm, David described an approach in which the aortic root wall is replaced by a straight tubular prosthesis thereby overcoming the shortcomings of lifelong anticoagulation. Currently, there are at least 5 modified procedures named David I to V [6] and although evidence on which one is better is lacking, a growing number of surgeons have been using David procedures for patients with connective tissue diseases. However, in the study of Marfan syndrome patients by Cameron and colleagues [7], the intrinsic leaflet structure in the graft without sinus structure deteriorated rapidly, undergoing prolapse and elongation possibly secondary to loss of tonicity and the free margin impacting the straight wall of the graft; long-time abrasion would therefore affect aortic valve leaflets. To address this limitation, in 2000, De Paulis and colleagues [8] introduced a graft which recreates sinuses and yields relatively favorable early outcomes: patients who underwent reimplantation with the Valsalva graft had a significantly smaller annular diameter, less bleeding, and less residual aortic insufficiency; however, the authors stressed that a larger patient cohort and longer follow-up was warranted.

Since 2005, our hospital has been using the VSSR procedure with De Paulis valsalva graft in patients with ascending aorta acute dissection or root aneurysm. As reported in this overview of our experience, one patient required reoperation for bleeding and there was only one death, yielding an operative mortality of 2.6 %, and no significant perioperative morbidity. One patient was converted to a Bentall procedure intraoperatively. Early outcomes of the 38 patients demonstrated no dilatation, and no significant aortic insufficiency or progression thereof.

Several experiences and observations are worth highlighting. First, in our experience, success rate improved after a learning curve for surgeons’ comprehensive understanding of anatomy and function of the aortic root and valve complex. Second, among the 38 patients, 16 who underwent VSSR procedure for acute aortic dissection, all survived without important events. Relative to the overall population studied, these patients were younger (41.8 ± 10.2 years), with a higher EuroScore II (23.4 ± 7.2), and smaller maximum aortic root diameter (44.7 ± 7.6 mm). The VSSR procedure appears to have been a good choice for these patients, however, further follow-up data are warranted.

Third, although VSSR procedure was reserved for cases with absence of severe aortic regurgitation, and presence of trileaflet aortic valve and normal cusps, it was performed in 2 cases or bicuspid aortic valve and 7 Marfan syndrome cases to honor patient’s desire for freedom from lifelong anticoagulation dependency. Although perioperative outcomes of VSSR were favorable, which could be related to the young age of the patients (40.7 ± 9.9; range, 24–55), long-term durability of VSSR is unknown in these cases because mean follow-up was 39.6 months (IQR, 3–71). If the conclusions of this study can be extended to these patient subsets, the valve and cusp repair strategies could further change the surgical approach in bicuspid aortic valve and Marfan syndrome cases [9–11].

This study has several limitations, including its small sample size, and limitations in statistical power to comparatively analyze subset of patients [12, 13]. Despite these limitations, this study with up to 9 years of follow-up documented excellent early and midterm outcomes in selected patients who underwent aortic root replacement for aneurysms.

## Conclusion

This procedure appears to be a safe and appropriate alternative method to mechanical composite valve graft root replacement in patients who wish to avoid anticoagulation dependency with warfarin.

## Abbreviations

IQR: interquartile range
VSSR: valve-sparing aortic root replacement
NYHA: New York Heart Association

## References

[1] David TE, Feindel CM (1992). An aortic valve-sparing operation for patients with aortic incompetence and aneurysm of the ascending aorta. J Thorac Cardiovasc Surg.

[2] Bentall H, De Bono A (1968). A technique for complete replacement of the ascending aorta. Thorax.

[3] Shrestha M, Baraki H, Maeding I, Fitzner S, Sarikouch S, Khaladj N (2012). Long-term results after aortic valve-sparing operation (David I). Eur J Cardiothorac Surg.

[4] Bonow RO, Carabello BA, Chatterjee K, de Leon AC, Faxon DP, Freed MD (2008). 2008 focused update incorporated into the ACC/AHA 2006 guidelines for the management of patients with valvular heart disease. J Am Coll Cardiol.

[5] Demers P, Miller DC (2004). Simple modification of “T. David-V” valve-sparing aortic root replacement to create graft pseudosinuses. Ann Thorac Surg.

[6] Shimizu H, Yozu R (2011). Valve-sparing aortic root replacement. Ann Thorac Cardiovasc Surg.

[7] Cameron DE, Alejo DE, Patel ND, Nwakanma LU, Weiss ES, Vricella LA (2009). Aortic root replacement in 372 Marfan patients: evolution of operative repair over 30 years. Ann Thorac Surg.

[8] De Paulis R, De Matteis GM, Nardi P, Scaffa R, Colella DF, Chiarello L (2000). A new aortic Dacron conduit for surgical treatment of aortic root pathology. Ital Heart J.

[9] Kvitting JP, Kari FA, Fischbein MP, Liang DH, Beraud AS, Stephens EH (2012). David valve-sparing aortic root replacement: equivalent mid-term outcome for different valve types with or without connective tissue disorder. J Thorac Cardiovasc Surg.

[10] David TE, Armstrong S, Manlhiot C, McCrindle BW, Feindel CM (2013). Long-term results of aortic root repair using the reimplantation technique. J Thorac Cardiovasc Surg.

[11] de Kerchove L, Boodhwani M, Glineur D, Poncelet A, Verhelst R, Astarci P (2009). Effects of preoperative aortic insufficiency on outcome after aortic valve-sparing surgery. Circulation.

[12] Aicher D, Kunihara T, Abou Issa O, Brittner B, Graber S, Schafers HJ (2011). Valve configuration determines long-term results after repair of the bicuspid aortic valve. Circulation.

[13] Volguina IV, Miller DC, LeMaire SA, Palmero LC, Wang XL, Connolly HM (2009). Valve-sparing and valve-replacing techniques for aortic root replacement in patients with Marfan syndrome: analysis of early outcome. J Thorac Cardiovasc Surg.

